# Transcriptional profiling reveals progeroid *Ercc1*^*-/Δ*^ mice as a model system for glomerular aging

**DOI:** 10.1186/1471-2164-14-559

**Published:** 2013-08-16

**Authors:** Bernhard Schermer, Valerie Bartels, Peter Frommolt, Bianca Habermann, Fabian Braun, Joachim L Schultze, Marianne Roodbergen, Jan HJ Hoeijmakers, Björn Schumacher, Peter Nürnberg, Martijn ET Dollé, Thomas Benzing, Roman-Ulrich Müller, Christine E Kurschat

**Affiliations:** 1 Department II of Internal Medicine and Center for Molecular Medicine Cologne, University of Cologne, Cologne, Germany; 2 Cologne Excellence Cluster on Cellular Stress Responses in Aging-Associated Diseases, University of Cologne, Cologne, Germany; 3 Systems Biology of Ageing Cologne, SyBaCol, University of Cologne, Cologne, Germany; 4 Cologne Center for Genomics, University of Cologne, Cologne, Germany; 5 Max Planck Institute for Biology of Ageing, Cologne, Germany; 6 Life and Medical Sciences Institute, University of Bonn, Bonn, Germany; 7 Laboratory for Health Protection Research, National Institute of Public Health and the Environment, Bilthoven, The Netherlands; 8 Department of Cell Biology and Genetics, Medical Genetics Centre, Erasmus MC, University Medical Centre Rotterdam, Rotterdam, The Netherlands

**Keywords:** Renal aging, Glomerular aging, Gene expression profiling, Microarray analysis, DNA damage, Nucleotide excision repair

## Abstract

**Background:**

Aging-related kidney diseases are a major health concern. Currently, models to study renal aging are lacking. Due to a reduced life-span progeroid models hold the promise to facilitate aging studies and allow examination of tissue-specific changes. Defects in genome maintenance in the *Ercc1*^*-/Δ*^ progeroid mouse model result in premature aging and typical age-related pathologies. Here, we compared the glomerular transcriptome of young and aged *Ercc1*-deficient mice to young and aged WT mice in order to establish a novel model for research of aging-related kidney disease.

**Results:**

In a principal component analysis, age and genotype emerged as first and second principal components. Hierarchical clustering of all 521 genes differentially regulated between young and old WT and young and old *Ercc1*^*-/Δ*^ mice showed cluster formation between young WT and *Ercc1*^*-/Δ*^ as well as old WT and *Ercc1*^*-/Δ*^ samples. An unexpectedly high number of 77 genes were differentially regulated in both WT and *Ercc1*^*-/Δ*^ mice (p < 0.0001). GO term enrichment analysis revealed these genes to be involved in immune and inflammatory response, cell death, and chemotaxis. In a network analysis, these genes were part of insulin signaling, chemokine and cytokine signaling and extracellular matrix pathways.

**Conclusion:**

Beyond insulin signaling, we find chemokine and cytokine signaling as well as modifiers of extracellular matrix composition to be subject to major changes in the aging glomerulus. At the level of the transcriptome, the pattern of gene activities is similar in the progeroid *Ercc1*^*-/Δ*^ mouse model constituting a valuable tool for future studies of aging-associated glomerular pathologies.

## Background

A dramatic increase in the elderly population is a predominant demographic change in industrialized countries; therefore aging-related diseases such as chronic kidney disease (CKD) are becoming an increasingly important medical issue for modern societies [1,2]. By 2030, 20% of the US population will be 65 years and older. In the European Union, the percentage of adults over 60 years of age will increase to 33% of the population by 2050 [2]. Due to aging-induced structural and functional tissue alterations as well as underlying diseases such as diabetes and arterial hypertension, the incidence of CKD will dramatically increase in the future [2,3]. CKD mleads to end-stage renal disease and is associated with an elevated overall as well as cardiovascular mortality, therefore constituting a considerable health and economic burden [4]. Understanding the mechanisms that contribute to the aging-associated decline in renal function will help to develop novel therapeutic and, more importantly, preventive strategies.

Kidneys of older patients exhibit morphological changes such as glomerulosclerosis, tubular atrophy, and interstitial fibrosis [2,3]. The expression of immune genes is increased in aged human kidneys [5], and senescent cells are known to exhibit a proinflammatory phenotype [6-8]. Altered levels of TGF-ß, EGF, IGF-1, and VEGF have been described in aging kidneys [9]. Tubulointerstitial inflammation, fibrosis, loss of kidney weight, and cortical thinning with a decrease of functional nephrons are well-known consequences of aging [2,7]. Starting at the age of 35 to 40 years, glomerular filtration rate (GFR) shows a decline of at least 0.75 ml/min/1.73 m^2^ per year and 6 ml/min/1.73 m^2^ per decade [10]. Aging-associated alterations also affect tubular and endocrine functions of the kidney.

DNA damage induced by oxidative stress plays a key role in the aging-associated decline of renal function [2]. Chronic renal failure has been liked to defects in DNA damage repair. DNA maintenance mechanisms such as interstrand crosslink (ICL) repair and nucleotide excision repair (NER) are essential to protect cells from DNA damage. Deficiencies in single NER proteins, specifically those implicated in the transcription-coupled repair (TCR) subpathway of NER, are known to induce severe progeroid syndromes. Many of these mutants display accelerated aging with shortened lifespan, progressive features of cachexia, kyphosis, retinal degeneration, renal abnormalities and neurological impairment. Among these disorders are Cockayne syndrome which is caused by mutations in ERCC6 (CSB) or ERCC8 (CSA), trichothiodystrophy (TTD) caused by mutations in ERCC2 (XPD), ERCC3 (XPB), or TTDA (GTF2H5) and XPF-ERCC1 progeria (XFE) caused by dysfunction of the XPF-ERCC1 heterodimer [11,12]. ERCC1 forms an endonuclease together with XPF that mediates 5’ incision of a damaged DNA strand. Furthermore, ERCC1/XPF is involved in ICL repair and homologous recombination [13,14]. *Ercc1* knockout mice (*Ercc1*^*-/-*^) are runted and suffer from severe progressive neurological abnormalities, kyphosis, and hepatic as well as renal nuclear abnormalities. They die before weaning at the age of 3 to 5 weeks due to hepatic insufficiency. A modified *Ercc1* knockout mouse model expressing a liver-specific *Ercc1* rescue transgene to prevent hepatic insufficiency shows an increased lifespan of 12 weeks. Interestingly, these animals develop proteinuria at 3 weeks of age and progress to end stage renal failure with uremic encephalopathy [15,16]. Thus, Ercc1 might play an important role in maintaining adequate renal function.

In order to extend lifespan of these mice to 6 months and to facilitate more extensive studies, Weeda et al. established a hemizygous *Ercc1-*deficient mouse model [17]. These mice harbor one loss-of-function allele and one hypomorphic allele that encodes a truncated protein lacking the last seven amino acids (*Ercc1*^*-/Δ*^). *Ercc1*^*-/Δ*^ mice exhibit tubular abnormalities, whereas glomerular function and morphology have not yet been investigated [13,14]. Since reduced Ercc1 function leads to a premature aging phenotype, we hypothesized that this mouse model might be suitable to study molecular mechanisms of glomerular aging. The premature aging phenotype holds the promise for facilitated specimen generation and simplification of study design compared to labor- and time-consuming aging studies in WT animals. Furthermore, animals with conditional *Ercc1* alleles are available to allow for cell-type-specific induction of aging in the kidney [18].

Therefore, we analyzed gene expression profiles of young and aged wild-type (WT) glomeruli and compared these profiles to young and old progeroid *Ercc1*^*-/Δ*^ glomeruli in order to examine the extent to which aging-related expression changes are recapitulated in the accelerated aging model. We now demonstrate that glomeruli of *Ercc1*-deficient mice reflect important aspects of physiological glomerular aging. Our analysis provides the first global view of the contribution of genome maintenance pathways to the prevention of glomerular aging.

## Results

### Identification of genes differentially expressed between young and old WT and *Ercc1*^*-/Δ*^ mice

**Figure 1:**
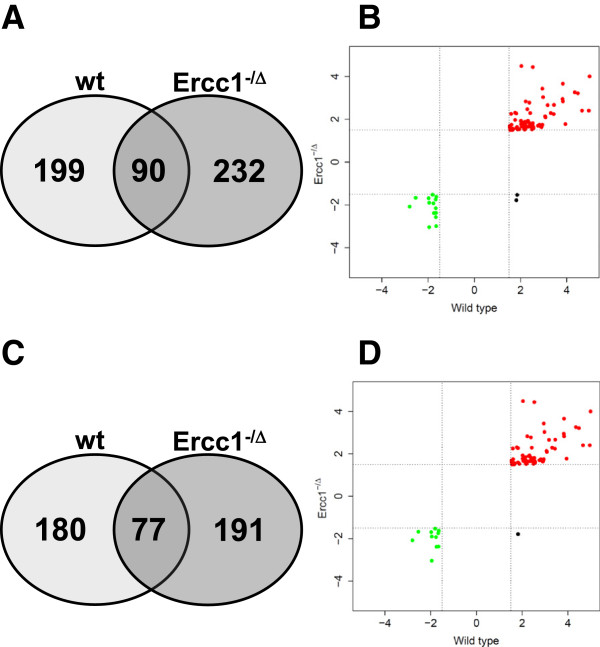
**Overlap of differentially expressed genes in aged WT and aged *Ercc1***^***-/Δ***^**mice. (A)** In aged WT glomeruli (light grey), 289 genes are differentially expressed (fold change ≥ 1.5). 322 genes are differentially expressed in aged *Ercc1*^*-/Δ *^glomeruli (dark grey). The intersection of the two groups contains 90 genes whereas only 3.2 would have been expected by chance alone (p < 0.0001, Fisher's exact test). **(B)** FC-FC plot of 90 genes representing the intersection of the two groups displayed in Figure 1A. **(C)** 73 genes differentially expressed in young WT mice between 4 and 14 weeks of age were considered to reflect kidney development and maturation and were subtracted from the dataset of both old WT and old *Ercc1*^*-/Δ *^glomeruli. 257 genes remained for aged WT glomeruli (light grey), and 268 genes for aged *Ercc1*^*-/Δ *^glomeruli (dark grey). 77 genes were differentially expressed in both datasets. Only 1.19 genes would have been expected to intersect by chance alone (p < 0.0001, Fisher's exact test). **(D)** FC-FC plot of 77 genes representing the intersection of the two groups displayed in Figure 1C.

*Ercc1*^*-/Δ*^ mice show pronounced premature aging with male mice achieving a median lifespan of 19 weeks and a maximal lifespan of 26 weeks and female mice reaching a median lifespan of 21 weeks and a maximal lifespan of 29 weeks respectively [13]. The median and maximal life span of their male WT siblings is 111 and 156 weeks [13]. To identify genes expressed in glomeruli that are associated with kidney aging, genome-wide transcriptome analyses were performed. 80% of the cohort is still alive at 14 wks for *Ercc1*^*-/Δ*^ and at 96 wks for WT animals. We hypothesized that 14 wk old WT animals were more suitable to be compared to 4 wk old *Ercc1*^*-/Δ*^ animals than 4 wk old WT animals. Since WT mice are weaned at the age of 3 weeks, a 4 wk old mouse is still adolescent whereas a 14 wk old mouse can be regarded as a young adult animal. Bioinformatic analysis revealed about 500 differentially expressed genes between age groups (fold change > 1.5, p < 0.05) in both WT and *Ercc1*^*-/Δ*^ mice. Gene expression changes of a set of genes that were significantly differentially expressed in the gene array experiments were quantitatively verified by quantitative PCR (see Additional file 1: Figure S1). We compared the expression pattern of glomerular tissue from young (4 wks) and old (14 wks) *Ercc1*^*-/Δ*^ mice to the expression pattern of glomerular tissue from young (14 wks) and old (96 wks) WT mice using whole-transcriptome microarrays. Using Venn diagrams, we found a surprisingly large overlap of 90 genes where only 3.2 genes would be expected by chance alone (p < 0.0001, Figure 1A). To address the question whether not only the genes themselves were identical but also the direction of regulation was the same in the two backgrounds we employed FC-FC plots (FC > 1.5) (Figure 1B). This analysis revealed 74 genes elevated at older age in WT and *Ercc1*^*-/Δ*^ mice and 14 genes decreased under both conditions. Only two genes were regulated in opposite directions between WT and *Ercc1*^*-/Δ*^ glomeruli.

In order to decrease the potential flaw in our gene expression analysis, we subtracted all genes regulated in WT glomeruli between 4 and 14 weeks of age from the initially created gene set of differentially expressed genes between 14 and 96 weeks of age (Figure 1C and 1D). These genes are hypothesized not to be regulated in an aging-dependent manner, but as a consequence of development and organ maturation (this gene set will from now on be referred to as the “maturation-adjusted differentially expressed gene set”). After subtraction, 77 regulated genes were shared by both WT and *Ercc1*^*-/Δ*^ glomeruli (Figure 1C). By chance alone, only 1.19 genes would be expected to intersect between both lists (p < 0.0001). Among these 77 genes, 65 were upregulated in both groups, and 11 genes were downregulated. Only a single gene was upregulated in WT and downregulated in *Ercc1*^*-/Δ*^ mice, shown by the FC–FC plot in Figure 1D (FC > 1.5).

### Principal component analysis and hierarchical clustering reveal similar aging-associated changes in gene expression of WT and *Ercc1*^*-/Δ*^ glomeruli

**Figure 2:**
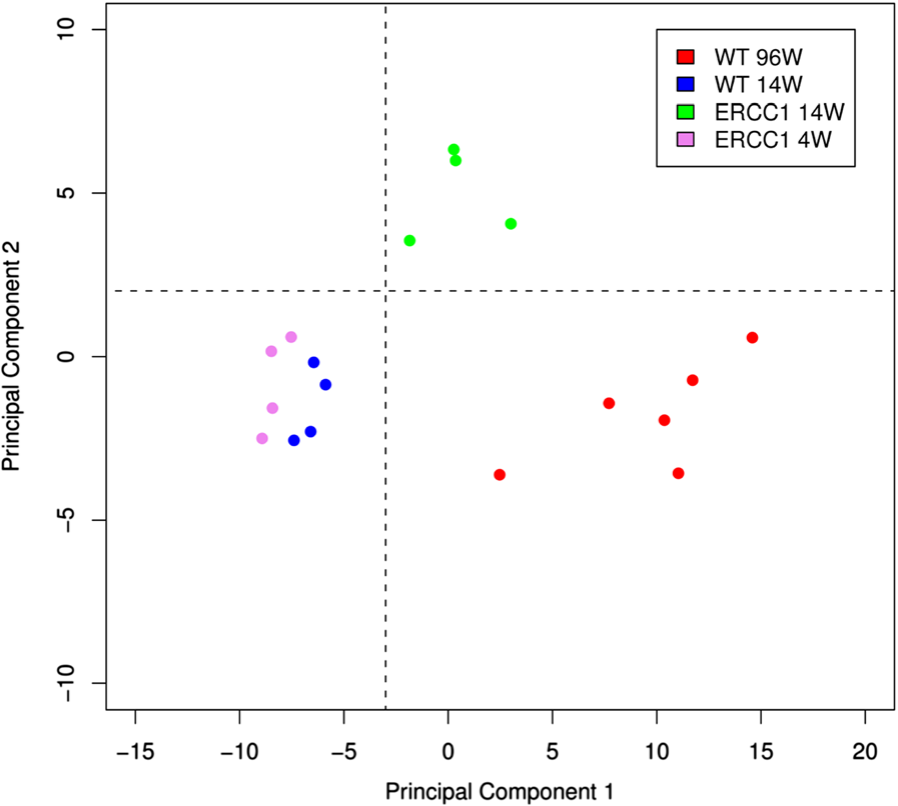
**Principal component analysis of young and old WT and *Ercc1***^***-/Δ ***^**mice.** This analysis takes into account all differentially regulated genes in WT mice after removal of early regulated genes. As the old and young samples are clearly separated along the x-axis, the first principal component (x-axis, eigenvalue 65.36) can be assumed to represent mouse age, indicating that a major part of the total variance in gene expression of the selected gene set can be explained by aging. On the y-axis, the major contributing factor to the second principle component can be assumed to be the genotypes (y-axis, eigenvalue 9.39).

We performed principal component analysis (PCA) on the transcriptomes of WT 14 and 96 wk old glomerular tissue and *Ercc1*^*-/Δ*^ 4 and 14 wk old glomerular tissue using the gene list of WT genes after subtraction of early differentially expressed genes in order to reduce the original high-dimensional data set into a set of PCs. Intriguingly, the first principal component mainly reflects mouse age (Figure 2, x-axis, eigenvalue of 65.36), while the second principal component can be explained by the different genotypes (Figure 2, y-axis, eigenvalue of 9.39). The eigenvalues essentially represent the variance explained by the principal components. The eigenvalue is almost 7 times higher for the first PC, indicating that the predominant factor driving transcriptional changes in this experiment is the animals’ age rather than the genotype. Our results confirm the similarity of transcriptional profiles of aged WT and aged *Ercc1*^*-/Δ*^ datasets versus young WT and young *Ercc1*^*-/Δ*^ datasets by showing a similar age-dependent shift on the level of the first principal component. A PCA using WT as well as *Ercc1*^*-/Δ*^ gene lists with similar results is shown in Additional file 2: Figure S2.

**Figure 3:**
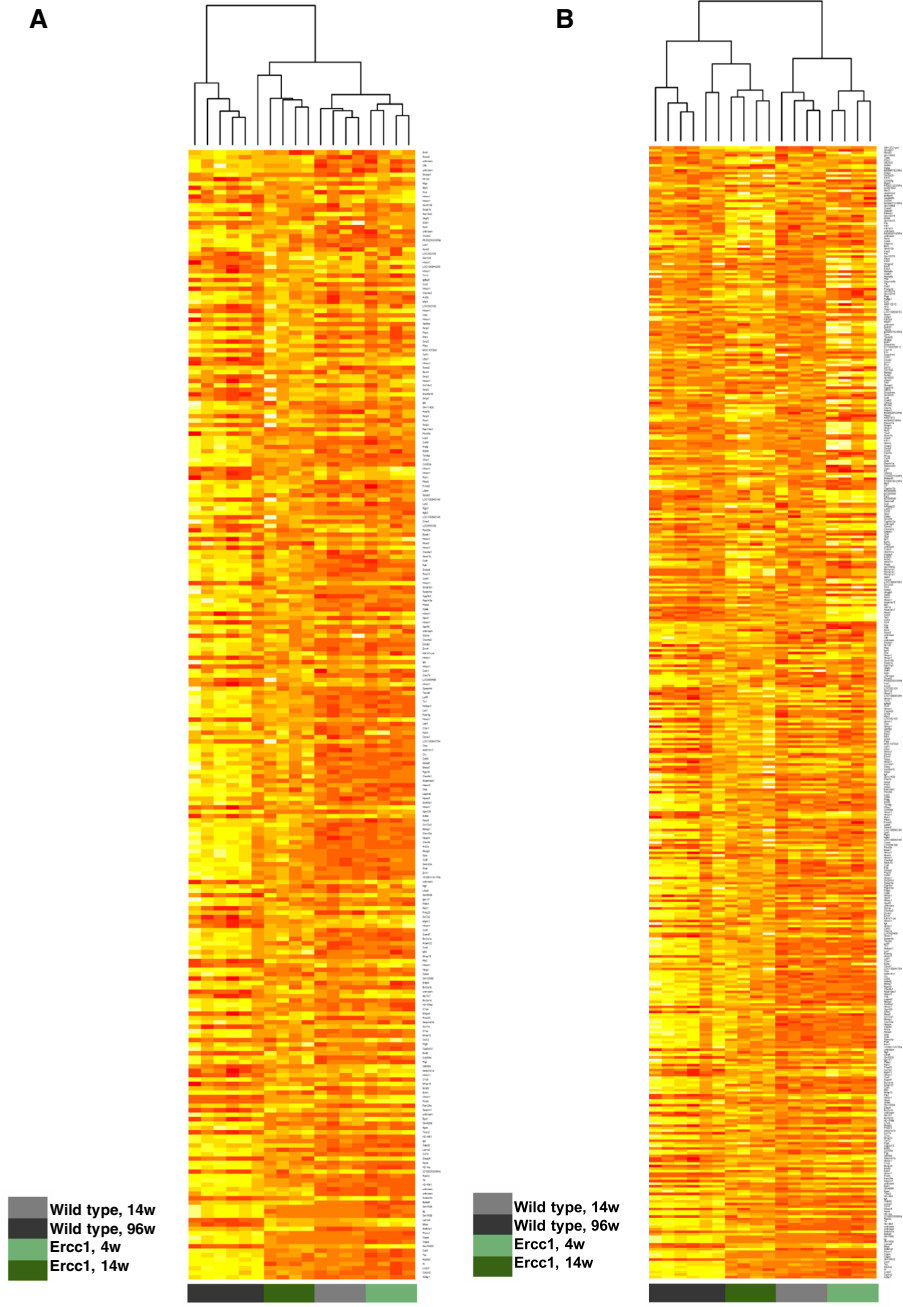
**Gene expression similarities of differentially regulated genes in WT and *Ercc1***^***-/Δ ***^**glomeruli. (A)** Hierarchical clustering of 257 genes differentially expressed in WT mice of 96 wks compared to WT 14 wks only. Glomerular genes differentially expressed in early mouse life between 4 and 14 weeks of age were considered to play a role in glomerular maturation and not in aging processes and thus were excluded from the dataset. This subset of genes mapped to aged WT mice (black), aged *Ercc1*^*-/Δ *^mice (dark green), 14 wks old WT mice (grey) and young *Ercc1*^*-/Δ *^mice (light green) is shown. Young WT samples share a subcluster with young *Ercc1*^*-/Δ *^samples, aged WT as well as *Ercc1*^*-/Δ *^samples show a distinct cluster. Upregulated genes are shown in orange, downregulated genes are displayed in yellow. **(B)** Hierarchical clustering of all 448 genes differentially regulated in WT mice of 96 wks compared to WT 14 wks as well as in *Ercc1*^*-/Δ *^mice of 14 wks compared to 4 wks. Prior to hierarchical clustering, glomerular genes differentially regulated in early mouse life between 4 and 14 weeks of age were subtracted from the dataset. Old wild-type mice (black) and old *Ercc1*^*-/Δ *^mice (dark green) shared a distinct subcluster. Furthermore, young wild-type mice (grey) and young *Ercc1*^*-/Δ *^mice (light green) share another distinct subcluster, reflecting the similarity of the sets of differentially regulated genes between WT and *Ercc1*^*-/Δ *^glomeruli in young as well as in old tissue. Upregulated genes are shown in orange, downregulated genes are displayed in yellow.

Presumably, the 257 differentially expressed genes of the maturation-adjusted gene set in WT mice (Figure 1C, 180 + 77) reflect physiological aging-associated transcriptional changes in our analysis. Unsupervised hierarchical clustering of this gene set using euclidean distance and complete linkage to analyze the similarity of expression profiles between aged WT and *Ercc1*^*-/Δ*^ glomeruli showed cluster formation of the young WT samples with young *Ercc1*^*-/Δ*^ glomerular samples. Old *Ercc1*^*-/Δ*^ glomerular samples show intermediate clustering (Figure 3A). Hierarchical clustering of the combined maturation-adjusted gene set of WT glomeruli and the differentially expressed gene list in *Ercc1*^*-/Δ*^ mice showed clearly, that old WT samples cluster with old *Ercc1*^*-/Δ*^ samples, and young WT samples cluster with young *Ercc1*^*-/Δ*^ samples (Figure 3B).

We repeated hierarchical cluster analysis of all regulated genes in old WT mice without maturation adjustment revealing the same association of samples (see Additional file 3: Figure S3A). Again, old samples clustered together, and young samples clustered together when using the whole list of all 521 differentially regulated genes in old WT mice as well as old *Ercc1*^*-/Δ*^ mice (Figure 1A) without maturation adjustment (see Additional file 4: Figure S3B).

These findings clearly show that 14 week old *Ercc1*^*-/Δ*^ glomeruli, on the transcriptional landscape, do not resemble glomeruli of WT mice of the same age, but rather those of aged 96 week old WT mice, pointing out that premature aging in *Ercc1*^*-/Δ*^ glomeruli shows features of physiological glomerular aging.

### The progeria mouse model *Ercc1*^*-/Δ*^ and WT aged mice share common molecular pathways

**Figure 4:**
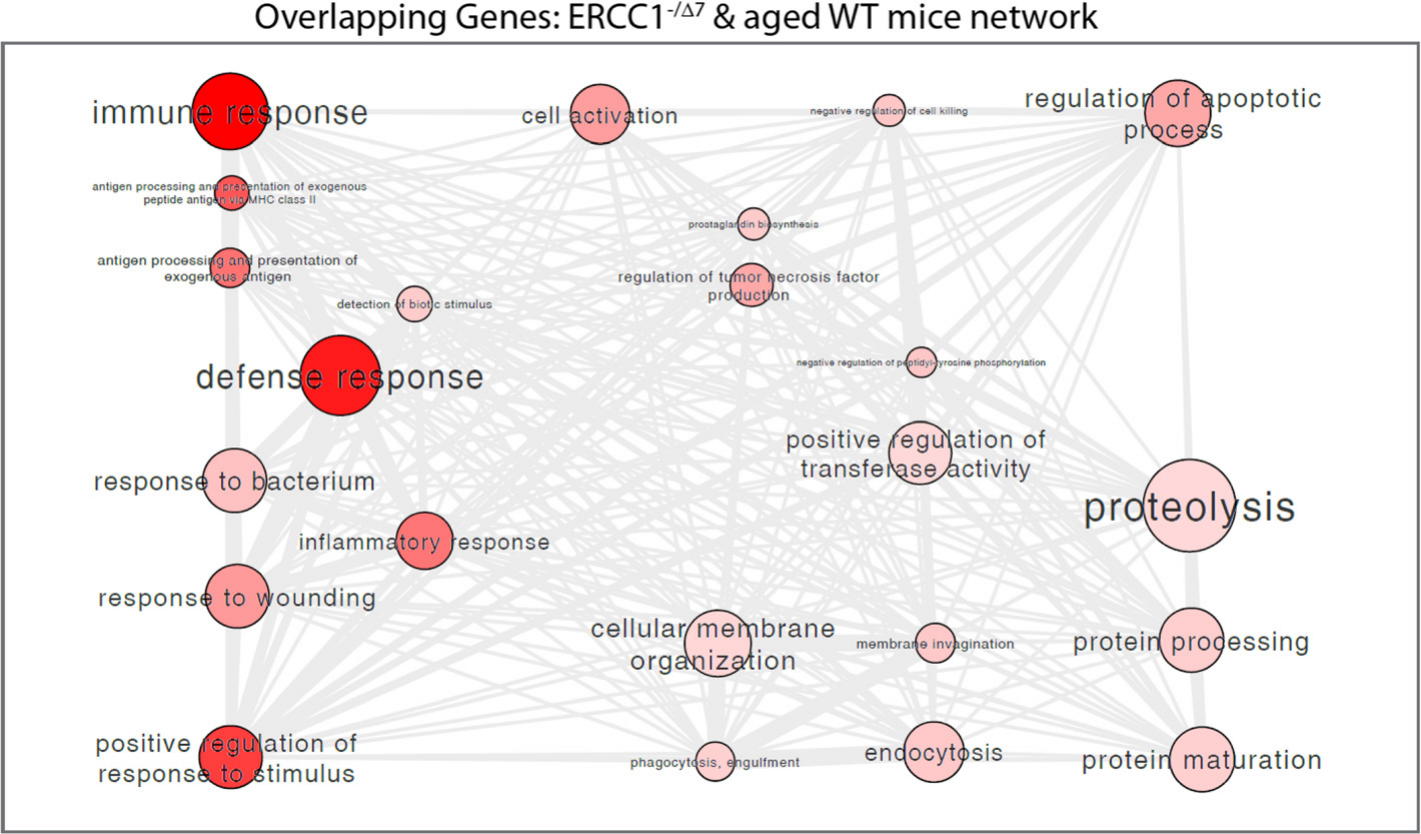
**Gene Ontology (GO) enrichments of WT and *Ercc1***^***-/Δ ***^**mice.** GO analysis of the 77 overlapping regulated genes in WT and *Ercc1*^*-/Δ*^ glomeruli demonstrate similarities between both datasets. The lower the p-value of term enrichment the darker the bubble color. Bubble sizes reflect the frequency of a respective GO term in the GO database. The lines represent hierarchical connections between GO terms. In both conditions, we find an enrichment of terms associated with immune response, defense response, proteolysis, endocytosis, and regulation of apoptotic processes. As expected, *Ercc1*^*-/Δ*^ samples additionally show a significant enrichment of terms associated with cell cycle/mitosis. The interactive graph was chosen from the REVIGO server.

To better understand whether common pathophysiologic mechanisms would explain these transcriptional changes in aged mice, we performed gene ontology (GO) analyses and visualized the data with Cytoscape [19] (Figure 4, Additional file 4: Figure S3A,B). Corresponding gene names are given in Additional file 5: Table S1 (WT) and Additional file 6: Table S2 (*Ercc1*^*-/Δ*^). Our results reveal significant functional similarities between aged WT and aged *Ercc1*^*-/Δ*^ glomeruli. Immune response genes, defense response genes, inflammatory response genes, response to wounding genes, genes regulating cell death, cell killing, cytolysis and apoptosis, chemotaxis, protein maturation and cation homeostasis were equally regulated in both aged glomerular tissues. GO analysis of only the 77 overlapping genes between WT and *Ercc1*^*-/Δ*^ is shown in Figure 4. Gene names are given in Additional file 7: Table S3. Most strikingly, immune response genes were regulated in both old WT and old *Ercc1*^*-/Δ*^ glomeruli. Some of these genes are already known to be associated with aging. Toll-like receptor 1 (TLR1) was found to exhibit increased expression in aged mouse brain [20], and chemokine receptor 5 (CCR5) has been associated with atherosclerosis and Alzheimer’s disease [21]. Protein tyrosine phosphatase receptor type C (PTPRC) shows increased expression levels in older healthy subjects [22]. CD74 expression is regulated in aged rats [23], and its expression is also increased in Pima Indians with diabetic nephropathy. In this model, CD74 expression was localized to podocytes [24].

The most striking difference between aged WT and aged *Ercc1*^*-/Δ*^ glomeruli was the prominent regulation of genes related to cell cycle, mitosis, organelle fission, and cell division (Additional file 4: Figure S3A,B). This finding is expected since Ercc1 plays a major role in genome maintenance processes such as nucleotide excision repair and DNA crosslink repair. Therefore, genes involved in cell cycle regulation and cell division are expected to be regulated in this model. Gene names of differentially expressed genes in WT, *Ercc1*^*-/Δ*^ and overlap genes are given in Additional file 5: Tables S1, Additional file 6: Table S2 and Additional file 7: Table S3. A list of genes encoding slit diaphragm proteins is given in Additional file 8: Table S4.

### Similarities and differences in gene regulation of aged WT and *Ercc1*^*-/Δ *^glomeruli by network analysis

**Figure 5:**
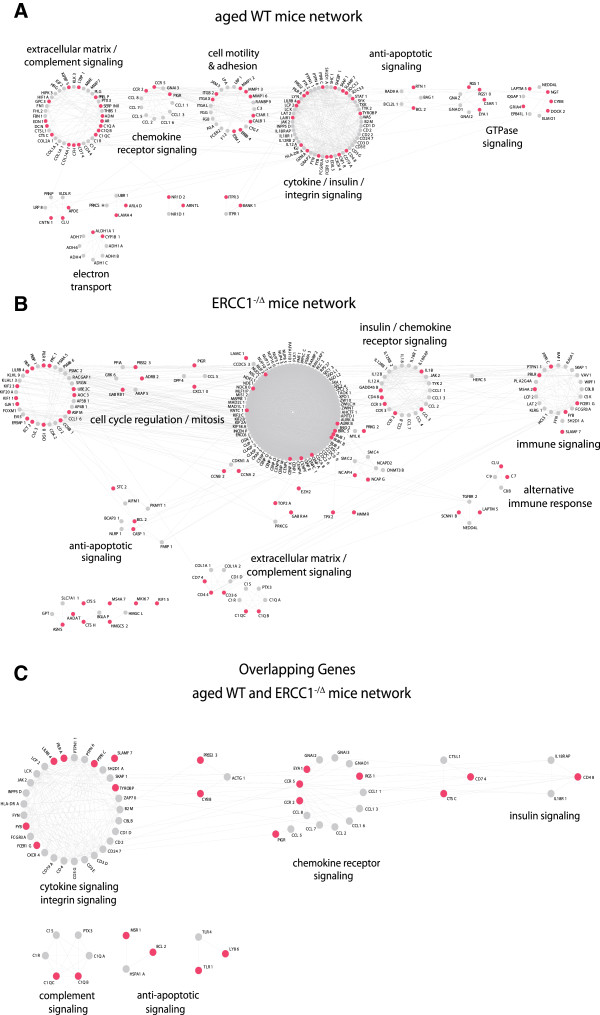
**Minimal state networks of WT and *Ercc1***^***-/Δ ***^**mice.** The graphs show the minimally connected interaction networks of wild type **(A)** and *Ercc1*^*-/Δ *^**(B)** mice. Pink circles indicate all genes that were differentially expressed. Grey circles indicate linker genes. Both networks contain functional modules associated with chemokine receptor signaling, insulin signaling, anti-apoptotic signaling as well as extracellular matrix and complement signaling. A major component of the *Ercc1*^*-/Δ *^network, as expected, consists of genes that are part of cell cycle regulation/mitosis. **(C)** Network analysis of only 77 overlapping regulated genes between WT and *Ercc1*^*-/Δ *^further illustrates similarities between WT and *Ercc1*^*-/Δ *^networks possibly reflecting aging processes.

To confirm similarities in gene regulation between aged WT and aged *Ercc1*^*-/Δ*^ glomeruli we performed network analyses. Mapping the list of differentially regulated genes on the human interactome with the NetBox software revealed additional networks similarly regulated in aged WT and aged *Ercc1*^*-/Δ*^ glomeruli (Figure 5A,B). Both figures display similarities regarding insulin signaling, chemokine/cytokine signaling, and extracellular matrix and complement signaling. A network analysis of only the 77 regulated overlapping genes in WT and *Ercc1*^*-/Δ*^ glomeruli also nicely shows the particular networks regulated in both aged glomerular datasets (Figure 5C).

As anticipated, the most striking difference between WT and *Ercc1*^*-/Δ*^ glomeruli was the differential regulation of genes responsible for cell cycle and cell division. Our DNA repair gene model revealed immune effector pathways as the main overarching principle of the aging phenotype.

## Discussion

We studied gene expression patterns of young and old WT mice glomeruli and compared these patterns to a mouse model of DNA damage and premature aging. Beyond pathways that are expected to play a role in glomerular aging such as apoptosis, proteolysis, and insulin signaling, we found gene regulatory networks involved in immune response, defense response, and inflammatory response mechanisms to be regulated in the aging glomerulus of both WT and *Ercc1*^*-/Δ*^ mice. These data clearly demonstrate that the progeroid *Ercc1*^*-/Δ*^ mouse model provides a helpful tool for future studies on aging-associated glomerular changes. However, addressing the detailed mechanism underlying glomerular aging in this strain will require additional studies that characterize the cell-specific expression of the genes identified within the glomerulus on the one hand. On the other hand – since we focused on transcriptional changes – an analysis of glomerular protein expression using proteomics holds the great potential to add to a better understanding of the consequences of the transcriptional changes on both protein levels and posttranslational modifications. On a functional level detailed histological examination of the mutant glomeruli will help to assess the role of immune cell invasion, cell death and alterations in the glomerular ultrastructure in the phenotype observed.

In our analysis, overlapping genes were part of insulin signaling, chemokine and cytokine signaling as well as extracellular matrix pathways. Moreover, our results xindicate that *Ercc1*^*-/Δ*^ mice – based on the transcriptional profiles – can be employed as a model of glomerular aging. Large-scale aging studies are significantly impaired by the difficulty in obtaining sufficient material due to the necessity of aging cohorts. Using prematurely aged mutant strains holds the promise to dramatically facilitate these studies. Nonetheless, it is crucial to determine whether these models are appropriate for examining the physiology of aging in a certain tissue. Furthermore, aging goes along with highly complex systemic changes. As for kidney deterioration, the cardiovascular system has tremendous impact on renal function and physiology. Using aged mouse cohorts or patient samples allows for studying the physiological processes of aging but as to tissue-specific and cell-intrinsic mechanisms, the data will always be impacted by the systemic confounders.

The aging process of an individual is characterized by the accumulation of progressive DNA lesions. Ercc1 is essential for genome stability, protecting cells against the consequences of a variety of endogenous and exogenous DNA damage through DNA repair. *Ercc1*-deficient mice exhibit a phenotype of premature aging [15,17,25]. In *Ercc1* knockout animals rescued for their severe lifespan-limiting, aging-related liver pathology, proteinuria and progressive chronic kidney disease are suggestive of a role of Ercc1 in glomerular cell biology [15]. ERCC1 is involved in human disease. A child with mutations in XPF/ERCC1 displayed a unique combination of progeroid symptoms and died from kidney failure at the age of 16 years [26]. A second child, compound heterozygous for two ERCC1 mutations, showed mild renal hypoplasia [27]. Due to the severe phenotype of *Ercc1* knockout mice, we decided to employ mice expressing the hypomorphic *Ercc1* allele (*Ercc1*^*-/Δ*^) as a putative model for glomerular aging biology [13,28]. When analyzing differentially expressed genes during the aging process in both WT and *Ercc1* mice the majority of regulated genes only exhibited a small change in expression levels as already described before [5,29-31]. The process of aging is probably not induced by the dysregulation of a small number of cellular pathways but rather a complex interplay between subtle changes in a variety of different cellular functions leading to senescence and cellular malfunction with age [32]. Studying only one pathway to elucidate aging-associated processes may thus be insufficient since one pathway might only contribute marginally to the whole picture of aging. Additionally, gene expression changes in aged tissues might also reflect adaptive responses.

Exploring associated GO term annotations, we identified striking similarities but also some differences between WT and *Ercc1*^*-/Δ*^. We were able to detect pathways that are expected to play a role in glomerular aging, such as regulation of apoptosis and proteolysis which has been previously described [33,34]. We also found that many differentially expressed genes are involved in immune response, defense response, and inflammatory response mechanisms. These pathways are also expected to play a role in aging processes, as already described by de Magalhães et al.[35] . Brink et al. compared aging processes of mouse brain, heart and kidney tissue and identified differentially expressed genes being part of immune and inflammatory response pathways, apoptosis and protein metabolism, such as CaspI, Irak3, Cd48, Dock2, and Icam1 [36]. In our study, Casp I and Cd48 were also differentially expressed in our aged glomerular samples. Analyzing human renal tissue, Rodwell et al. revealed that genes differentially displayed with age were expressed in B cells, T cells and neutrophils [5]. It can be speculated that immune response pathways influence the aging process in WT as well as in *Ercc1*^*-/Δ7*^ glomerular tissue. Whether these genes are expressed by cells having invaded the glomerulus or by resident glomerular cells such as mesangial cells, endothelial cells or podocytes has not been determined yet.

In a transcriptional analysis of human whole kidney samples at three different age groups by Melk et al., genes involved in extracellular matrix turnover, energy metabolism and mitochondrial function were differentially expressed in old kidneys [30]. These genes were not significantly regulated in our aged glomerular samples possibly reflecting the differences between whole kidney tissue and glomerular tissue, or expressing species differences. In our study, we identified additional genes involved in matrix turnover. OSF-2/periostin was significantly downregulated in old glomerular tissue samples. Periostin is known to play a role in glomerulosclerosis and renal interstitial fibrosis [37], both entities are associated with aging. Interestingly, these authors also identified genes involved in non-specific inflammatory responses to play a role during the aging process. Chronic organ injury is known to induce a focal increase in B cell content. This infiltration might occur due to increased fibrosis and atrophy and/or altered cytokine expression profiles of aged resident renal cells. Since this finding has been observed in several studies [38], elucidating the mechanism of immune cell infiltration with aging is important for future studies on tissue aging processes.

As expected, differences between WT and *Ercc1*^*-/Δ*^ mice could also be demonstrated in this analysis due to the underlying genetic defect in *Ercc1*^*-/Δ*^ mice. Mainly genes involved in cell cycle and cell division were differentially expressed in *Ercc1*^*-/Δ*^ mice, but not in WT mice. Nevertheless, our data demonstrate a significant overlap of aging-associated genes in the two different mouse models. In our analysis, gene expression was not dominated by genotype-specific changes that might override subtle age-related changes in gene expression. In old *Ercc1*^*-/Δ*^ mice, changes owing to the aging process were more prominent than those brought forth by the underlying genotype. This is not always found in other studies. As an example, Amador-Noguez at el. analyzed gene expression profiles from young versus old liver samples of long-lived Ames dwarf and Little mice. In their study, they observed that gene expression changes due to the underlying genotype were more dramatic than changes due to age itself [31].

The identification of the *Ercc1*^*-/Δ*^ background as a genetic tool for the induction of glomerular aging holds a great potential for future studies of glomerular aging. First, use of this model – due to the premature aging phenotype at 14 weeks of age - highly facilitates this research which is often impaired by difficulties in maintaining large cohorts of aged mice. Second, it is now within reach to induce aging in a cell-specific manner by using conditional alleles of *Ercc1*[18]. Thus it will be possible to dissect glomerulus-intrinsic aging from systemic effects due to cardiovascular changes and aging of the renal microvasculature on the one hand. On the other hand cell-specific aging within the glomerulus can now be addressed to examine the hypothesis that assumedly post-mitotic cells as the podocytes are major contributors to aging-related pathologies.

Aging is a complex process. A better understanding of the molecular biology involved in aging-related pathologies is the pre-requisite for developing novel ways of tackling aging-related disease. We already know that interventions such as dietary restriction or certain drugs such as rapamycin can increase lifespan and organismal stress resistance. Nevertheless – in order to take advantage of these phenomena in the clinical setting – the underlying molecular mechanisms will need to be elucidated.

## Conclusions

Our results identify insulin signaling, apoptosis, proteolysis and pathways involved in immune response, defense response and inflammatory response mechanisms to be involved in glomerular aging. Their overlap provides evidence that molecular aging processes in *Ercc1*-deficient mouse glomeruli are comparable to those in WT glomeruli. In the future, *Ercc1*^*-/Δ*^ mice can be used as a model system to study aging of the glomerular filtration system.

## Methods

### Mice

*Ercc1*^*+/Δ*^ FVB mice and *Ercc1*^*+/-*^, pUR288(LacZ)^+/+^ C57BL/6 mice were used as breeding pairs to generate wild-type *Ercc1*^*+/+*^, pUR288^+/-^ control and *Ercc1*^*-/Δ7*^, pUR288^+/-^ repair deficient mice in a genetically uniform hybrid C57BL/6-FVB background [13]. Genotyping of these mice was performed as previously reported [13].

All experiments were conducted according to federal and institutional guidelines and have been approved by the Committee on Animal Experimentation (IACUC) of the Antonie van Leeuwenhoek terrain (DEC-Alt) in Bilthoven, NIH/NIA 1PO1 AG 17242.

Housing was done under specific pathogen-free (SPF) conditions. Three-monthly monitoring according to FELASA suggestions was performed, including six additional viruses [13,39]. Adult mice were housed in groups of less than five animals separated by genotype and gender. Mice received CRM pelleted breeder and maintenance diet that was 25 kGy irradiated (Special Diet Services, Witham, UK), and water *ad libitum*. For our studies, only male mice were used. 4 WT mice at 4 weeks of age, 4 WT mice at 14 weeks of age, and 6 WT mice at 96 weeks of age were available for isolation of glomeruli. In the *Ercc1*^*-/Δ*^ group, 4 mice were sacrificed after 4 weeks, another 4 mice after 14 weeks. 96 weeks for WT and 14 weeks for *Ercc1*^*-/Δ*^ represent the age at which 80% of the cohort was still alive.

### Isolation of glomeruli

The abdominal aorta and both kidneys were dissected. Left kidneys were perfused via the renal artery with 2 × 10^8^ Dynabeads (Dynabeads M-450 Tosylactivated, Life Technologies, Invitrogen, Darmstadt, Germany), diluted in 10 ml of Hank’s buffered salt solution (HBSS, Sigma-Aldrich Chemie GmbH, Taufkirchen, Germany), then minced in pieces and digested in 1 ml HBSS containing 1 mg collagenase (Sigma Aldrich Chemie GmbH, Taufkirchen, Germany) and 100 U DNase (Roche Diagnostics Deutschland GmbH, Mannheim, Germany) for 30 min in a thermoshaker (Eppendorf AG, Hamburg, Germany) at 37°C. Further processing to isolate glomeruli using a magnetic particle concentrator (MPC, Invitrogen Dynal AS, Oslo, Norway) was conducted as previously described [40]. Washed glomeruli were resuspended in 750 μl Qiazol (Qiagen, Hilden, Germany), frozen on dry ice and stored at -80°C until further processing.

### Preparation of RNA

Total RNA was extracted and purified using commercial homogenization (Bio 101 FastPrep FP120-120 V, Savant, Midland, MI, USA) and the RNeasy kit (Qiagen, Hilden, Germany).

### Microarray hybridization

RNA was reverse transcribed with the Applause WT-Amp ST RNA Amplification System (NuGen Technologies, Inc., San Carlos, CA, USA) in accordance to the manufacturer’s protocol. Resultant cDNA probes were labeled with the Encore Biotin Module (NuGen Technologies, Inc.) and hybridized to the Affymetrix GeneChip Mouse Gene 1.0 ST Array according to the manufacturer’s instructions. Finally, chips were scanned with a GeneChip 3,000 6G scanner.

### Quantitative RT-PCR

Regulation of high-scoring genes from our microarray analyses was re-assessed through quantitative real-time PCR using SYBR green on an ABI 7900 HT thermocycler (Applied Biosystems, Life Technologies Cooperation, Carlsbad, CA, USA). Expression levels were normalized to housekeeping genes B2M and PGK, and calculated with the comparative threshold cycle (Ct) method as described previously [41]. Primer sequences are available upon request. Data is given as Additional file 1: Figure S1A for WT and Additional file 1: Figure S1B for *Ercc1*^*-/Δ*^ glomeruli.

### Affymetrix microarray data analyses

Raw data (CEL files) were processed using the robust multi-array average (RMA) algorithm and quantile normalization with the Affymetrix Power Tools, version 1.12.0, and platform-specific library files [42]. Differential gene expression was analyzed using descriptive statistics (fold change) and Tukey's method for pairwise comparisons between any 2 of the 3 groups (wild type) or Student's *t*-test (*Ercc1*^*-/Δ*^ mice). Genes were prioritized by statistical evidence. In order to create candidate lists for differential gene expression between two conditions, we used all genes regulated at least 1.5-fold where differential expression was significant at level 0.05. Type I error inflation was ignored because the p-values were used to prioritize the list rather than being interpreted in a confirmatory sense. For multiple hypothesis testing for the genes further discussed we used the Bonferroni method. Genes differentially regulated in early WT mouse life (between 4 and 14 wks of age) were considered to reflect glomerular development and maturation rather than aging. Therefore, these 73 early regulated genes were subtracted from old WT and old *Ercc1*^*-/Δ*^ gene lists. These modified lists were used to perform PCA, GO mapping and network analyses (Figures 2, 4, 5, respectively).

For statistical analysis, in particular for principal component analysis (PCA) and hierarchical clustering, we used the related functions of the R system for statistical computing, version 2.9.2. The genes overlapping between the lists for *Ercc1* and wild type were displayed in two-by-two tables (not shown) and the number of genes present in both lists was assessed using Fisher's exact test. The number of overlapping genes as expected by chance alone was calculated from the marginal totals as usual in a two-by-two table analysis. All microarray data reported in this study are described in accordance with MIAME guidelines and have been deposited in the National Center for Biotechnology Information Gene Expression Omnibus (GEO, http://www.ncbi.nlm.nih.gov/geo/) public repository. The data sets supporting the results of this article are available in the GEO public repository at http://www.ncbi.nlm.nih.gov/geo/query/acc.cgi?acc=GSE43061.

### Enrichment analysis and term visualization

Annotation of differentially expressed genes, as well as enrichment analysis using standard parameters was done using the DAVID server (http://david.abcc.ncifcrf.gov/) [43]. Visualization of GO term enrichment was performed using REVIGO (http://revigo.irb.hr/) with standard parameters [44]. Cytoscape was used for visualization [19].

### Network analysis

Network analysis was done using NetBox [45]. Mouse genes were mapped on the human interactome provided with the NetBox software, which includes interaction data from HPRD, Reactome and the Cancer NCI Pathways. Genes that were either not present or could not be mapped between species were pruned from the hit lists. Visualization was performed using Cytoscape [19].

### Availability of supporting data

The data sets supporting the results of this article are available in the GEO public repository at http://www.ncbi.nlm.nih.gov/geo/query/acc.cgi?acc=GSE43061.

## Competing interests

The authors declare that they have no competing interest.

## Authors’ contributions

BS participated in the design of the study, conducted the experiments, and edited the manuscript. VB was responsible for statistical analyses, interpretation of data, and drafting of the manuscript. PF performed bioinformatic analyses. BH performed pathway and network analyses. JLS and FB participated in statistical analysis, interpreted the data and revised the manuscript. MR and METD designed the study, generated the mice, and supervised the study. JHJH was involved in editing the manuscript and in generation of the mouse mutant. BS revised the manuscript; PN was responsible for hybridization of microarrays. TB supervised the study and revised the manuscript. RUM was responsible for data interpretation and editing of the manuscript. CEK interpreted the data, supervised the study, and drafted the manuscript. All authors read and approved the final manuscript.

## Authors’ information

Roman-Ulrich Müller and Christine E Kurschat are senior authors.

## Supplementary Material

Additional file 1: Figure S1 Microarray validation of randomly selected regulated genes by quantitative RT-PCR. The regulation of 10 randomly selected genes from the microarray analysis of WT glomeruli **(A)** and *Ercc1*^*-/Δ *^glomeruli **(B)** was validated by using quantitative real-time PCR. All genes investigated were regulated in the same direction in microarray datasets and in qRT-PCR. Expression levels of housekeeping genes B2M and PGK were set to 1 (dotted line). Click here for file

Additional file 2: Figure S2 Principal component analysis of young and old WT and *Ercc1*^*-/Δ *^mice without subtraction of early differentially expressed genes. PCA including all differentially expressed genes in WT mice without subtracting early differentially expressed genes shows that the first principal component can be interpreted as mouse age (x-axis, eigenvalue 71.80), indicating that a substantial part of the total variance in gene expression of the selected gene set can be explained by aging. Also in this analysis, mouse genotype emerges as the second principal component (y-axis, eigenvalue 10.94). Click here for file

Additional file 3: Figure S3A Gene expression similarities of differentially expressed genes in WT and *Ercc1*^*-/Δ*^.glomeruli without subtraction of early regulated genes. **(A)** Hierarchical clustering of all 289 genes differentially expressed in WT mice of 96 wks compared to WT 14 wks without subtraction of early differentially regulated genes (Figure 1A); aged WT mice (black), aged *Ercc1*^*-/Δ *^mice (dark green), 14 wks old WT mice (grey) and young *Ercc1*^*-/Δ *^mice (light green). Young WT samples share a subcluster with young *Ercc1*^*-/Δ *^samples, aged WT as well as *Ercc1*^*-/Δ *^samples show a distinct cluster. (B) Hierarchical clustering of all 521 genes differentially expressed in WT mice of 96 wks compared to WT 14 wks as well as in *Ercc1*^*-/Δ *^mice of 14 wks compared to 4 wks (Figure 1A). As already shown in Figure 3B, aged WT glomerular samples (black) cluster together with aged *Ercc1*^*-/Δ *^samples (dark green), and young WT samples (grey) cluster together with young *Ercc1*^*-/Δ *^samples (light green) indicating that age is the major factor contributing to the similarity of the transcriptional profiles among samples in contrast to the underlying genotype. Click here for file

Additional file 4: Figure S3B GO terms that are enriched in the lists of differentially regulated genes found in wild type **(A)** and *Ercc1*^*-/Δ *^**(B)** glomeruli are shown. The lower the p-value of term enrichment the darker the bubble color. Bubble sizes reflect the frequency of a respective GO term in the GO database. In both conditions, we find an enrichment of terms associated with immune response, defense response, proteolysis, endocytosis, and regulation of apoptotic processes. Click here for file

Additional file 5: Table S1 GO enrichment analysis of genes differentially expressed between young (14 wks) and old (96 wks) WT glomerular samples. Click here for file

Additional file 6: Table S2 GO enrichment analysis of genes differentially expressed between young (4 wks) and old (14 wks) *Ercc1*^*-/Δ *^glomerular samples. Click here for file

Additional file 7: Table S3 GO enrichment analysis of overlapping genes between Table 1 and Table 2. Click here for file

Additional file 8: Table S4 List of genes encoding slit diaphragm proteins. Click here for file
