# Supplementary figures and images for: Transcriptional profiling reveals progeroid Ercc1-/Δ mice as a model system for glomerular aging
Source: BMC Genomics. 2013 Aug 16;14:559. doi: 10.1186/1471-2164-14-559 (PMC3751413; doi:10.1186/1471-2164-14-559)

# Suppl. Figure 1

A.

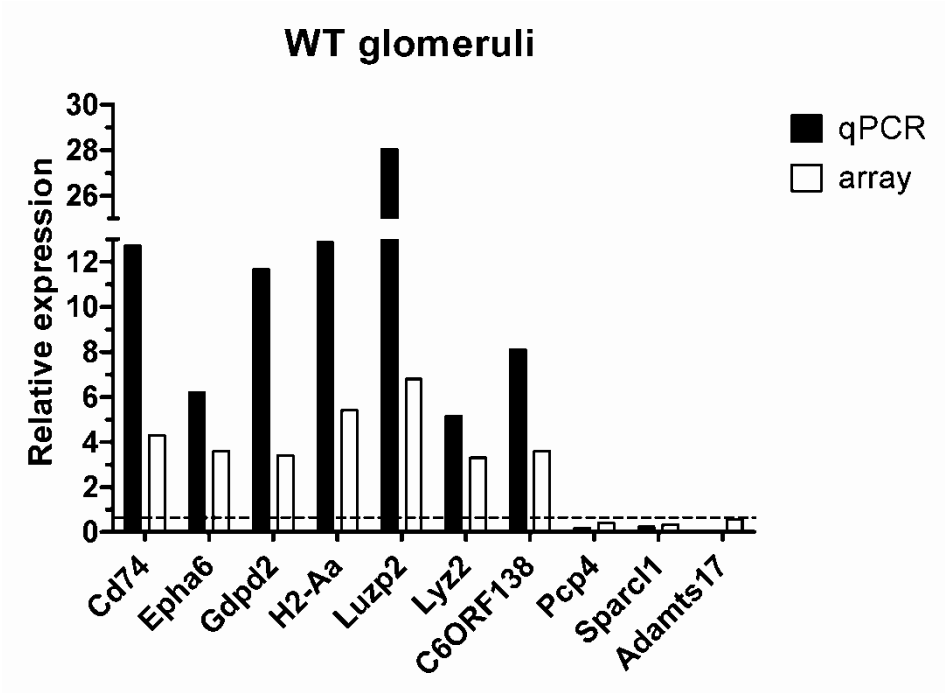

B.

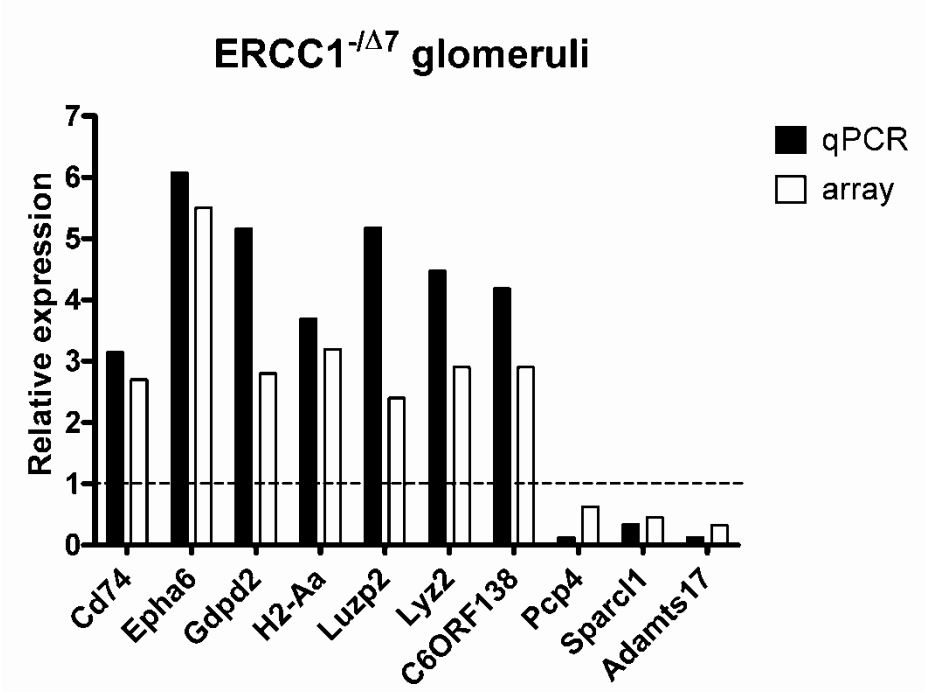

Supplement: Additional file 1: Figure S1 — Microarray validation of randomly selected regulated genes by quantitative RT-PCR. The regulation of 10 randomly selected genes from the microarray analysis of WT glomeruli (A) and Ercc1-/Δ glomeruli (B) was validated by using quantitative real-time PCR. All genes investigated were regulated in the same direction in microarray datasets and in qRT-PCR. Expression levels of housekeeping genes B2M and PGK were set to 1 (dotted line). [file 1471-2164-14-559-S1.pdf]

# Suppl. Figure 2

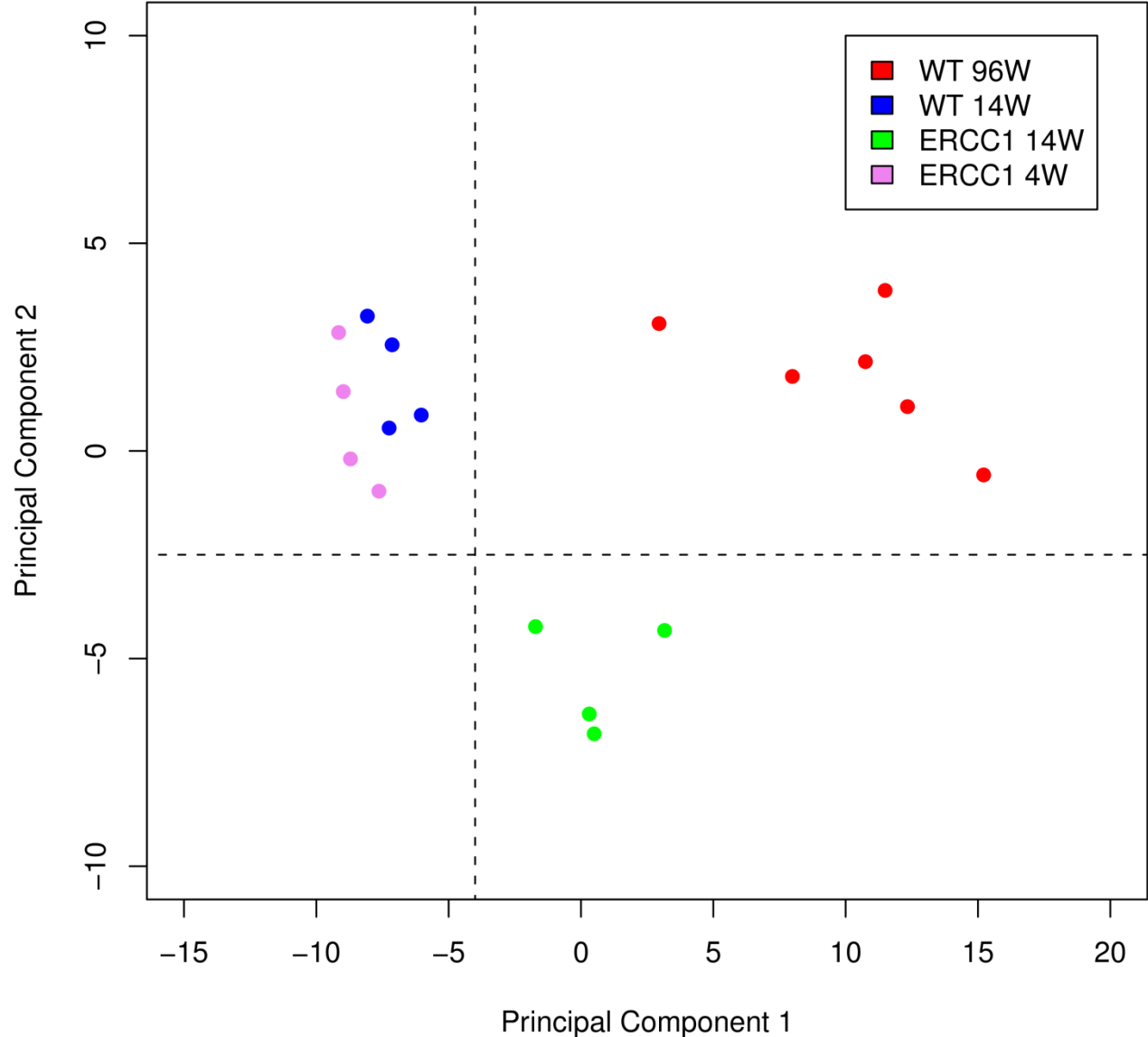

Supplement: Additional file 2: Figure S2 — Principal component analysis of young and old WT and Ercc1-/Δ mice without subtraction of early differentially expressed genes. PCA including all differentially expressed genes in WT mice without subtracting early differentially expressed genes shows that the first principal component can be interpreted as mouse age (x-axis, eigenvalue 71.80), indicating that a substantial part of the total variance in gene expression of the selected gene set can be explained by aging. Also in this analysis, mouse genotype emerges as the second principal component (y-axis, eigenvalue 10.94). [file 1471-2164-14-559-S2.pdf]

**Suppl. Figure 3 A**

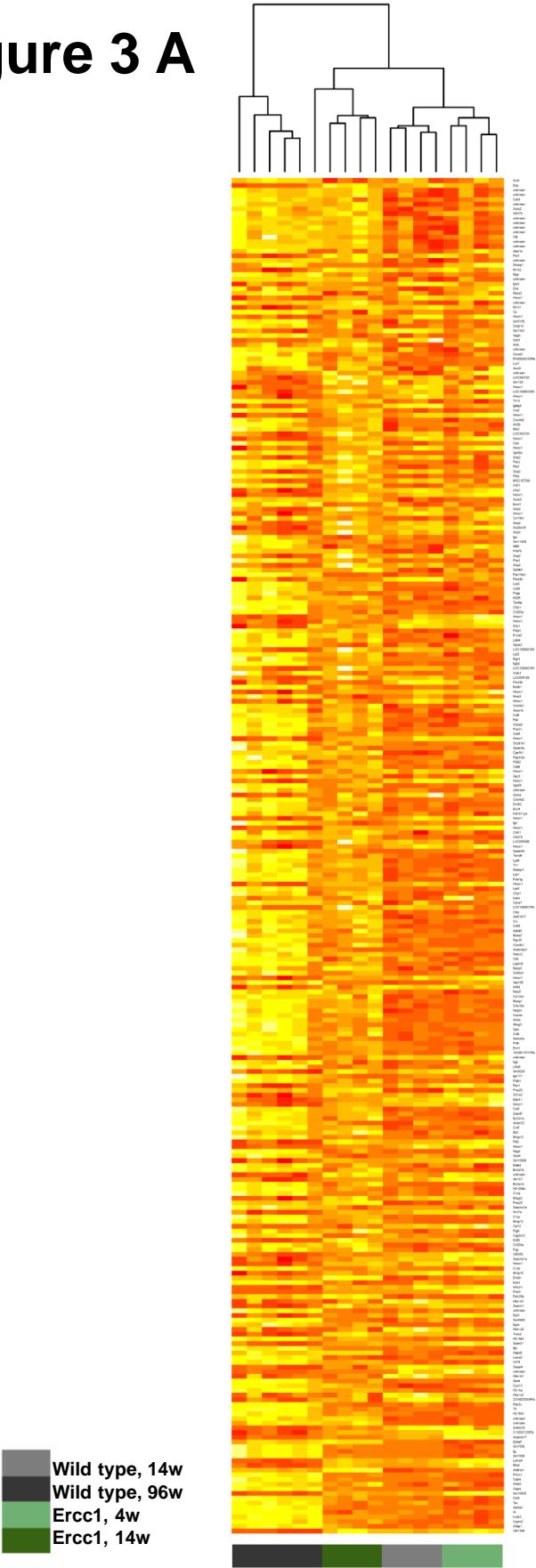

Supplement: Additional file 3: Figure S3A — Gene expression similarities of differentially expressed genes in WT and Ercc1-/Δ.glomeruli without subtraction of early regulated genes. (A) Hierarchical clustering of all 289 genes differentially expressed in WT mice of 96 wks compared to WT 14 wks without subtraction of early differentially regulated genes (Figure 1A); aged WT mice (black), aged Ercc1-/Δ mice (dark green), 14 wks old WT mice (grey) and young Ercc1-/Δ mice (light green). Young WT samples share a subcluster with young Ercc1-/Δ samples, aged WT as well as Ercc1-/Δ samples show a distinct cluster. (B) Hierarchical clustering of all 521 genes differentially expressed in WT mice of 96 wks compared to WT 14 wks as well as in Ercc1-/Δ mice of 14 wks compared to 4 wks (Figure 1A). As already shown in Figure 3B, aged WT glomerular samples (black) cluster together with aged Ercc1-/Δ samples (dark green), and young WT samples (grey) cluster together with young Ercc1-/Δ samples (light green) indicating that age is the major factor contributing to the similarity of the transcriptional profiles among samples in contrast to the underlying genotype. [file 1471-2164-14-559-S3.pdf]

# Suppl. Figure 3 B

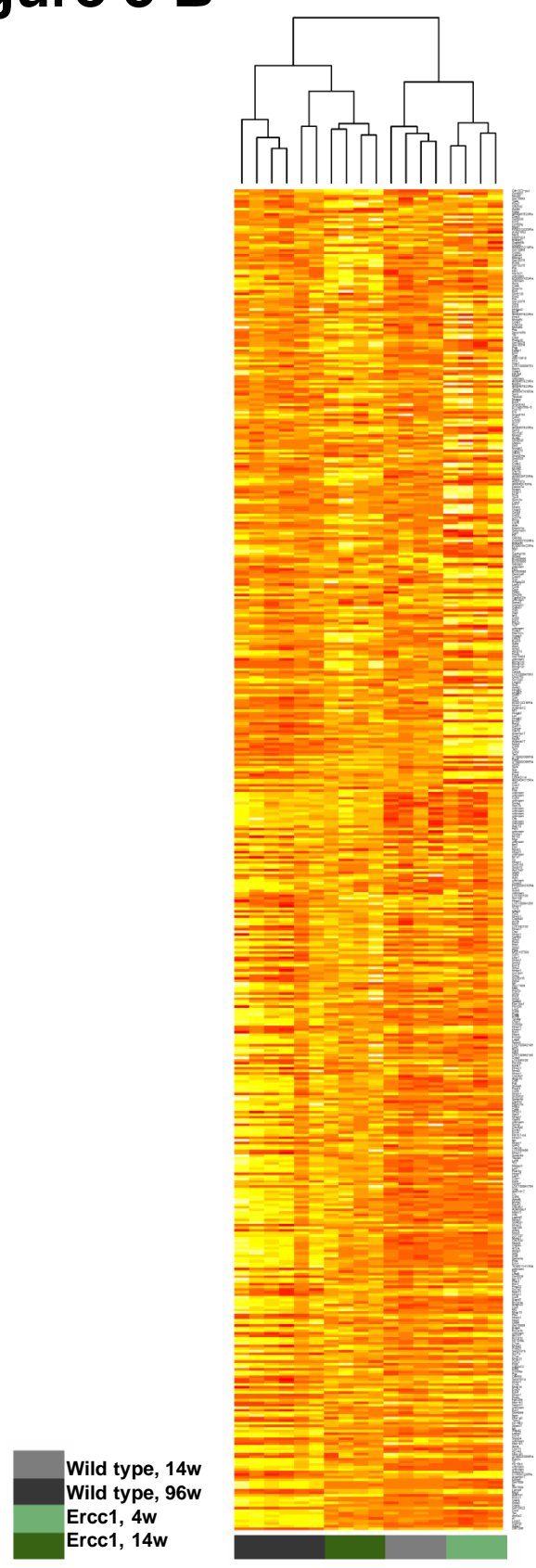

Supplement: Additional file 4: Figure S3B — GO terms that are enriched in the lists of differentially regulated genes found in wild type (A) and Ercc1-/Δ (B) glomeruli are shown. The lower the p-value of term enrichment the darker the bubble color. Bubble sizes reflect the frequency of a respective GO term in the GO database. In both conditions, we find an enrichment of terms associated with immune response, defense response, proteolysis, endocytosis, and regulation of apoptotic processes. [file 1471-2164-14-559-S4.pdf]
