# Supplementary material for: Transcriptional profiling reveals progeroid Ercc1-/Δ mice as a model system for glomerular aging
Source: BMC Genomics. 2013 Aug 16;14:559. doi: 10.1186/1471-2164-14-559 (PMC3751413; doi:10.1186/1471-2164-14-559)
Supplement: Additional file 5: Table S1 — GO enrichment analysis of genes differentially expressed between young (14 wks) and old (96 wks) WT glomerular samples. [file 1471-2164-14-559-S5.pdf]

Suppl. table 1: genes differentially expressed in aged WT mice

GO BP Term	# of Genes	P-value	Genes
immune response	26	1.70E-11	LY86, TLR1, CCL9, C1QC, CD74, CCL6, GP49A, CFH, FCER1G, CLEC4A2, SPON2, PTPRC, TLR13, IGJ, H2-AB1, CLEC4N, C1QA, C1QB, CCR5, CD300A, LILRB4, H2-EB1, CCR2, H2-AA, CLEC7A, H2-DMA, LCP1
leukocyte activation	16	8.95E-09	FYB, PTPRC, TLR1, ITGB2, CD74, CD48, DOCK2, CD86, BCL2A1D, ITGAX, SLC7A2, FCER1G, BANK1, H2-DMA, LCP1, LCP2
defense response	21	4.13E-08	PTPRC, LYZ2, LYZ1, C5AR1, LY86, TLR13, TLR1, ITGB2, C1QC, TRF, CD74, C1QA, C1QB, SERPINA1B, CCR5, SLC7A2, CCR2, CFH, FCER1G, H2-AA, CLEC7A
cell activation	16	4.26E-08	FYB, PTPRC, TLR1, ITGB2, CD74, CD48, DOCK2, CD86, BCL2A1D, ITGAX, SLC7A2, FCER1G, BANK1, H2-DMA, LCP1, LCP2
antigen processing and presentation of peptide antigen via MHC class II	6	4.62E-07	H2-EB1, H2-AA, FCER1G, H2-AB1, H2-DMA, CD74
antigen processing and presentation of exogenous peptide antigen via MHC class II	6	4.62E-07	H2-EB1, H2-AA, FCER1G, H2-AB1, H2-DMA, CD74
inflammatory response	14	6.58E-07	LY86, TLR13, TLR1, ITGB2, C1QC, TRF, C1QA, C1QB, SERPINA1B, CCR5, SLC7A2, CCR2, CFH, CLEC7A
antigen processing and presentation of peptide or polysaccharide antigen via MHC class II	6	1.20E-06	H2-EB1, H2-AA, FCER1G, H2-AB1, H2-DMA, CD74
antigen processing and presentation of exogenous peptide antigen	6	3.36E-06	H2-EB1, H2-AA, FCER1G, H2-AB1, H2-DMA, CD74
T cell activation	10	3.41E-06	CD48, PTPRC, CD86, DOCK2, BCL2A1D, ITGAX, ITGB2, H2-DMA, CD74, LCP1
positive regulation of response to stimulus	12	3.95E-06	C1QA, C1QB, PTPRC, BCL2A1D, EYA1, CFH, H2-AA, FCER1G, EDA2R, CLEC7A, H2-DMA, C1QC
antigen processing and presentation of exogenous antigen	6	9.41E-06	H2-EB1, H2-AA, FCER1G, H2-AB1, H2-DMA, CD74
positive regulation of immune response	10	1.25E-05	C1QA, C1QB, PTPRC, BCL2A1D, CFH, H2-AA, FCER1G, CLEC7A, H2-DMA, C1QC
response to wounding	15	1.62E-05	LY86, TLR1, TLR13, ITGB2, C1QC, TRF, C1QA, C1QB, SERPINA1B, CCR5, SLC7A2, CCR2, CFH, CLEC7A, LCP1
antigen processing and presentation of peptide antigen	6	2.93E-05	H2-EB1, H2-AA, FCER1G, H2-AB1, H2-DMA, CD74
activation of immune response	8	3.17E-05	C1QA, C1QB, PTPRC, BCL2A1D, CFH, FCER1G, CLEC7A, C1QC
lymphocyte activation	11	3.17E-05	CD48, PTPRC, CD86, DOCK2, BCL2A1D, ITGAX, ITGB2, BANK1, H2-DMA, CD74, LCP1
immunoglobulin mediated immune response	7	4.88E-05	C1QA, C1QB, H2-AA, FCER1G, H2-DMA, C1QC, CD74
immune effector process	9	5.20E-05	C1QA, C1QB, PTPRC, CFH, H2-AA, FCER1G, H2-DMA, C1QC, CD74
B cell mediated immunity	7	5.83E-05	C1QA, C1QB, H2-AA, FCER1G, H2-DMA, C1QC, CD74
positive thymic T cell selection	4	6.02E-05	PTPRC, DOCK2, H2-DMA, CD74
positive regulation of immune system process	11	6.03E-05	C1QA, C1QB, PTPRC, BCL2A1D, CFH, H2-AA, FCER1G, CLEC7A, H2-DMA, C1QC, CD74
positive T cell selection	4	1.27E-04	PTPRC, DOCK2, H2-DMA, CD74
lymphocyte mediated immunity	7	1.40E-04	C1QA, C1QB, H2-AA, FCER1G, H2-DMA, C1QC, CD74
taxis	8	1.44E-04	C3AR1, DOCK2, C5AR1, CCL9, FCER1G, ITGB2, SEMA3A, CCL6
chemotaxis	8	1.44E-04	C3AR1, DOCK2, C5AR1, CCL9, FCER1G, ITGB2, SEMA3A, CCL6
T cell proliferation	5	1.93E-04	PTPRC, CD86, DOCK2, ITGAX, ITGB2
acute inflammatory response	7	2.00E-04	C1QA, C1QB, SERPINA1B, SLC7A2, CFH, C1QC, TRF
humoral immune response	6	2.43E-04	C1QA, C1QB, CCR2, IGJ, CFH, C1QC
adaptive immune response	7	2.44E-04	C1QA, C1QB, H2-AA, FCER1G, H2-DMA, C1QC, CD74
adaptive immune response based on somatic recombination of immune receptors built from immunoglobulin superfamily domains	7	2.44E-04	C1QA, C1QB, H2-AA, FCER1G, H2-DMA, C1QC, CD74
leukocyte mediated immunity	7	3.34E-04	C1QA, C1QB, H2-AA, FCER1G, H2-DMA, C1QC, CD74
positive regulation of developmental process	10	4.12E-04	PTPRC, VEGFC, MSR1, SOCS2, CLU, H2-AA, ITGB2, H2-DMA, CD74, NGF
thymic T cell selection	4	4.64E-04	PTPRC, DOCK2, H2-DMA, CD74
myeloid leukocyte activation	5	4.65E-04	FYB, SLC7A2, TLR1, FCER1G, LCP2
positive regulation of cell differentiation	9	5.00E-04	PTPRC, VEGFC, MSR1, SOCS2, CLU, H2-AA, H2-DMA, CD74, NGF
innate immune response	7	8.92E-04	C1QA, C1QB, TLR13, TLR1, CFH, CLEC7A, C1QC
locomotory behavior	10	9.11E-04	C3AR1, DOCK2, C5AR1, NPAS3, CCL9, FCER1G, ITGB2, SEMA3A, CALB1, CCL6
lymphocyte proliferation	5	9.40E-04	PTPRC, CD86, DOCK2, ITGAX, ITGB2
