# Supplementary material for: Transcriptional profiling reveals progeroid Ercc1-/Δ mice as a model system for glomerular aging
Source: BMC Genomics. 2013 Aug 16;14:559. doi: 10.1186/1471-2164-14-559 (PMC3751413; doi:10.1186/1471-2164-14-559)
Supplement: Additional file 6: Table S2 — GO enrichment analysis of genes differentially expressed between young (4 wks) and old (14 wks) Ercc1-/Δ glomerular samples. [file 1471-2164-14-559-S6.pdf]

Suppl. table 2: genes differentially expressed in aged Ercc1$^{-/\Delta}$ mice

GO BP Term	# of Genes	P-value	Genes
M phase of mitotic cell cycle	19	5.30E-11	CDK1, KIF11, NUF2, KNTC1, CENPF, NUSAP1, BIRC5, ANLN, NDC80, AURKB, UBE2C, CCNB1, NCAPH, CCNB2, CDCA2, BUB1, BUB1B, CCNA2, ASPM
nuclear division	18	3.30E-10	CDK1, KIF11, NUF2, KNTC1, NUSAP1, ANLN, NDC80, BIRC5, AURKB, UBE2C, CCNB1, NCAPH, CCNB2, CDCA2, BUB1, BUB1B, CCNA2, ASPM
mitosis	18	3.30E-10	CDK1, KIF11, NUF2, KNTC1, NUSAP1, ANLN, NDC80, BIRC5, AURKB, UBE2C, CCNB1, NCAPH, CCNB2, CDCA2, BUB1, BUB1B, CCNA2, ASPM
cell division	21	5.12E-10	CDK1, CKS1B, KIF11, PRC1, NUF2, KNTC1, NUSAP1, BIRC5, ANLN, NDC80, AURKB, UBE2C, CCNB1, NCAPH, CCNB2, CDCA2, BUB1, BUB1B, CCNA2, TOP2A, ASPM
organelle fission	18	5.81E-10	CDK1, KIF11, NUF2, KNTC1, NUSAP1, ANLN, NDC80, BIRC5, AURKB, UBE2C, CCNB1, NCAPH, CCNB2, CDCA2, BUB1, BUB1B, CCNA2, ASPM
M phase	21	5.81E-10	CDK1, KIF11, MKI67, NUF2, KNTC1, TPX2, NUSAP1, CENPF, BIRC5, ANLN, NDC80, AURKB, UBE2C, CCNB1, NCAPH, CCNB2, CDCA2, BUB1, BUB1B, CCNA2, ASPM
mitotic cell cycle	19	2.24E-09	CDK1, KIF11, NUF2, KNTC1, CENPF, NUSAP1, BIRC5, ANLN, NDC80, AURKB, UBE2C, CCNB1, NCAPH, CCNB2, CDCA2, BUB1, BUB1B, CCNA2, ASPM
cell cycle phase	21	7.45E-09	CDK1, KIF11, MKI67, NUF2, KNTC1, TPX2, NUSAP1, CENPF, BIRC5, ANLN, NDC80, AURKB, UBE2C, CCNB1, NCAPH, CCNB2, CDCA2, BUB1, BUB1B, CCNA2, ASPM
cell cycle process	21	1.50E-07	CDK1, KIF11, MKI67, NUF2, KNTC1, TPX2, NUSAP1, CENPF, BIRC5, ANLN, NDC80, AURKB, UBE2C, CCNB1, NCAPH, CCNB2, CDCA2, BUB1, BUB1B, CCNA2, ASPM
inflammatory response	15	1.17E-06	CCL3, LY86, TLR1, F8, C1QC, CXCL10, C1QB, CCL12, SERPINA3N, P2RX7, CCR5, CD44, CCR2, IL1B, CLEC7A
defense response	21	1.19E-06	PTPRC, CCL3, LYZ2, LY86, TLR1, F8, C1QC, CD74, CXCL10, C1QB, CCL12, SERPINA3N, P2RX7, CCR5, CD44, CCR2, FCER1G, IL1B, H2-AA, CLEC7A, DEFB1
response to wounding	18	2.28E-06	CCL3, PLEK, LY86, TLR1, F8, GJA1, C1QC, CXCL10, C1QB, CCL12, SERPINA3N, P2RX7, CCR5, CD44, CCR2, IL1B, CLEC7A, SCNN1B
cell cycle	24	3.34E-06	CKAP2, CDK1, CKS1B, KIF11, PRC1, MKI67, NUF2, TPX2, KNTC1, NUSAP1, CENPF, BIRC5, ANLN, NDC80, AURKB, UBE2C, CCNB1, NCAPH, CCNB2, CDCA2, BUB1, BUB1B, CCNA2, ASPM
immune response	19	3.45E-05	PTPRC, CCL3, LY86, TLR1, H2-AB1, C1QC, CD74, CXCL10, C1QB, CCL12, CCR5, GP49A, LILRB4, CCR2, CD300LG, FCER1G, IL1B, H2-AA, CLEC7A, DEFB1
cytokinesis	5	3.62E-04	PRC1, NUSAP1, BIRC5, ANLN, AURKB
positive regulation of developmental process	11	4.35E-04	PTPRC, ADRB2, P2RX7, CD36, MSR1, CLU, H2-AA, GJA1, IL1B, CD74, IFI204
positive regulation of response to stimulus	10	6.54E-04	C1QB, PTPRC, P2RX7, EYA1, H2-AA, FCER1G, IL1B, EDA2R, CLEC7A, C1QC
