# Supplementary material for: Transcriptional profiling reveals progeroid Ercc1-/Δ mice as a model system for glomerular aging
Source: BMC Genomics. 2013 Aug 16;14:559. doi: 10.1186/1471-2164-14-559 (PMC3751413; doi:10.1186/1471-2164-14-559)
Supplement: Additional file 7: Table S3 — GO enrichment analysis of overlapping genes between Table 1 and Table 2. [file 1471-2164-14-559-S7.pdf]

Suppl. table 3: overlap of genes differentially expressed in aged WT and aged Ercc1$^{-/\Delta}$ mice

GO BP Term	# of Genes	P-value	Genes
immune response	13	1.21E-07	PTPRC, LY86, TLR1, H2-AB1, C1QC, CD74, C1QB, CCR5, GP49A, CCR2, LILRB4, H2-AA, FCER1G, CLEC7A
defense response	12	6.41E-07	C1QB, PTPRC, LYZ2, CCR5, LY86, CCR2, TLR1, H2-AA, FCER1G, CLEC7A, C1QC, CD74
positive regulation of response to stimulus	8	5.60E-06	C1QB, PTPRC, EYA1, H2-AA, FCER1G, EDA2R, CLEC7A, C1QC
antigen processing and presentation of exogenous peptide antigen via MHC class II	4	2.70E-05	H2-AA, FCER1G, H2-AB1, CD74
antigen processing and presentation of peptide antigen via MHC class II	4	2.70E-05	H2-AA, FCER1G, H2-AB1, CD74
antigen processing and presentation of peptide or polysaccharide antigen via MHC class II	4	4.63E-05	H2-AA, FCER1G, H2-AB1, CD74
antigen processing and presentation of exogenous peptide antigen	4	8.37E-05	H2-AA, FCER1G, H2-AB1, CD74
immunoglobulin mediated immune response	5	8.91E-05	C1QB, H2-AA, FCER1G, C1QC, CD74
B cell mediated immunity	5	1.01E-04	C1QB, H2-AA, FCER1G, C1QC, CD74
immune effector process	6	1.06E-04	C1QB, PTPRC, H2-AA, FCER1G, C1QC, CD74
positive regulation of immune system process	7	1.15E-04	C1QB, PTPRC, H2-AA, FCER1G, CLEC7A, C1QC, CD74
positive regulation of immune response	6	1.51E-04	C1QB, PTPRC, H2-AA, FCER1G, CLEC7A, C1QC
antigen processing and presentation of exogenous antigen	4	1.53E-04	H2-AA, FCER1G, H2-AB1, CD74
lymphocyte mediated immunity	5	1.85E-04	C1QB, H2-AA, FCER1G, C1QC, CD74
inflammatory response	7	1.86E-04	C1QB, CCR5, LY86, CCR2, TLR1, CLEC7A, C1QC
adaptive immune response based on somatic recombination of immune receptors built from immunoglobulin superfamily domains	5	2.72E-04	C1QB, H2-AA, FCER1G, C1QC, CD74
adaptive immune response	5	2.72E-04	C1QB, H2-AA, FCER1G, C1QC, CD74
activation of immune response	5	2.98E-04	C1QB, PTPRC, FCER1G, CLEC7A, C1QC
antigen processing and presentation of peptide antigen	4	3.00E-04	H2-AA, FCER1G, H2-AB1, CD74
leukocyte mediated immunity	5	3.40E-04	C1QB, H2-AA, FCER1G, C1QC, CD74
immune response-regulating cell surface receptor signaling pathway	4	5.92E-04	PTPRC, SH2D1B1, FCER1G, CLEC7A
immune response-regulating signal transduction	4	9.67E-04	PTPRC, SH2D1B1, FCER1G, CLEC7A
