# Supplementary material for: Transcriptional profiling reveals progeroid Ercc1-/Δ mice as a model system for glomerular aging
Source: BMC Genomics. 2013 Aug 16;14:559. doi: 10.1186/1471-2164-14-559 (PMC3751413; doi:10.1186/1471-2164-14-559)
Supplement: Additional file 8: Table S4 — List of genes encoding slit diaphragm proteins. [file 1471-2164-14-559-S8.pdf]

Suppl. table 4: List of genes encoding slit diaphragm proteins

Differential expression of genes encoding slit diaphragm proteins		
Glomeruli		
Gene	WT	Ercc1 -/delta
Podocin	-1,1	-1,0
Synaptopodin2	1,0	1,1
CD2AP	-1,1	-1,0
ZO-1	-1,3	-1,0
Fyn	-1,3	-1,0
Nephrin	-1,2	1,0
Neph1	-1,3	-1,1
Neph2	-1,0	-1,1
